# Ladder Mechanisms of Ion Transport in Prussian Blue Analogues

**DOI:** 10.1021/acsami.1c20910

**Published:** 2021-12-22

**Authors:** Johan Nordstrand, Esteban Toledo-Carrillo, Sareh Vafakhah, Lu Guo, Hui Ying Yang, Lars Kloo, Joydeep Dutta

**Affiliations:** † Functional Materials, Applied Physics Department, School of Engineering Sciences, KTH Royal Institute of Technology, AlbaNova Universitetscentrum, 106 91 Stockholm, Sweden; ‡ Pillar of Engineering Product Development, Singapore University of Technology and Design, Singapore 487372; § Applied Physical Chemistry, Department of Chemistry, KTH Royal Institute of Technology, SE-100 44 Stockholm, Sweden

**Keywords:** capacitive deionization, finite element, multiscale modeling, Prussian blue analogues, self-consistent mean-field theory, quantum chemistry

## Abstract

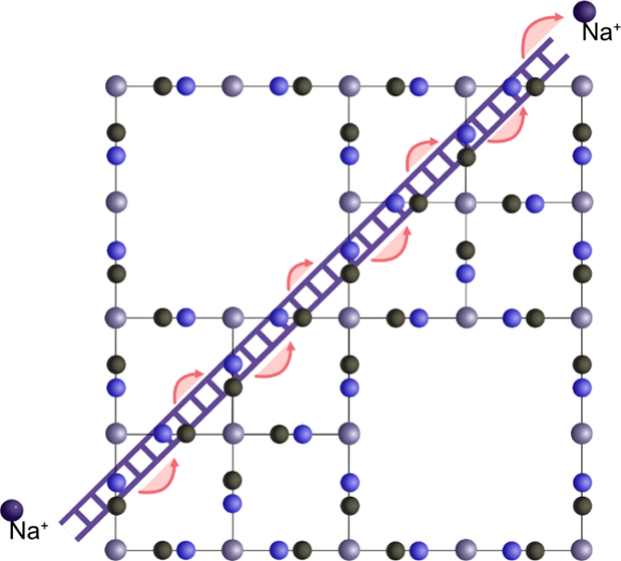

Prussian blue (PB) and its analogues (PBAs) are drawing attention as promising materials for sodium-ion batteries and other applications, such as desalination of water. Because of the possibilities to explore many analogous materials with engineered, defect-rich environments, computational optimization of ion-transport mechanisms that are key to the device performance could facilitate real-world applications. In this work, we have applied a multiscale approach involving quantum chemistry, self-consistent mean-field theory, and finite-element modeling to investigate ion transport in PBAs. We identify a cyanide-mediated ladder mechanism as the primary process of ion transport. Defects are found to be impermissible to diffusion, and a random distribution model accurately predicts the impact of defect concentrations. Notably, the inclusion of intermediary local minima in the models is key for predicting a realistic diffusion constant. Furthermore, the intermediary landscape is found to be an essential difference between both the intercalating species and the type of cation doping in PBAs. We also show that the ladder mechanism, when employed in multiscale computations, properly predicts the macroscopic charging performance based on atomistic results. In conclusion, the findings in this work may suggest the guiding principles for the design of new and effective PBAs for different applications.

## Introduction

Energy storage is a crucial step toward a sustainable society. While lithium-ion batteries have been in use for many years,^1^ researchers are beginning to search for other sustainable alternatives.^2^ Sodium-ion batteries are thus drawing attention due to the high abundance and availability of the element on earth. Of the various options being perused for improving the efficiency of sodium-ion batteries, Prussian blue analogues (PBAs)^3,4^ are promising alternatives for overcoming the challenges of switching from lithium to sodium ions as energy carriers.^1^ Interestingly, a wide variety of other applications exist for PBAs as well, such as energy storage,^5^ decontamination of cesium,^6−9^ sensing,^10^ Fenton reactions,^11^ carbon dioxide (CO_2_) capture,^12^ biomedicine,^13,14^ and capacitive deionization (CDI).^15−28^

**Figure 1:**
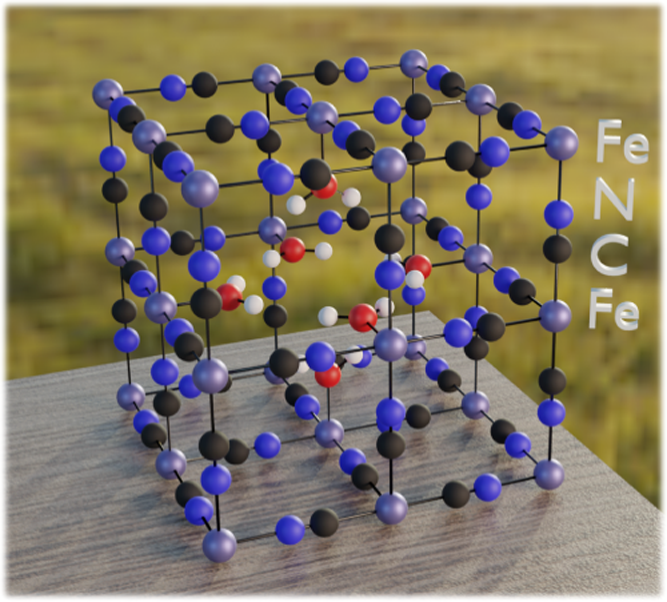
Unit cell of iron Prussian blue as modeled in this work, containing a central vacancy and coordinated water molecules (Fe_4_[Fe(CN)_6_]_3_·6H_2_O).

Intercalation diffusion rates are often identified as a limiting factor in battery materials.^29^ Thus, PBAs represent good materials for making sodium-ion batteries because their wide interstitial pathways permit effective sodium-ion transport even though the sodium ions are larger than lithium ions.^1^ Additionally, they are inexpensive and relatively easy to produce.^1^ PBAs constitute a wide family of materials^30−33^ of the ideal composition M[M′(CN)_6_],^34^ where M and M′ commonly are iron (Fe) but can also define a wide variety of di/trivalent atoms, such as copper (Cu), cobalt (Co), and manganese (Mn), among others.^4^ One major source of uncertainty is that the crystal structure of PBAs shows a high density of vacancy defects, up to 33%,^35^ which complicates the ion-transport processes inside the crystalline material.^1^ For instance, the ideal structural formula for the so-called insoluble Prussian blue is Fe_4_[Fe(CN)_6_]_3_, showing that the core crystal should have at least one-quarter (1/4) of the [Fe(CN)_6_] complexes missing (Figure 1).^4^ The complexity and diversity of the PBA crystal composition make modeling crucial for understanding and predicting the material and ultimately the device performance.

Simulations using quantum-chemistry calculations represent a powerful tool for understanding material properties at an atomic level.^36^ Previous studies of PBAs have highlighted the importance of a detailed material structure for modeling ionic transport processes. For instance, the cyanide (CN^–^) skeleton defining the inner walls of PBA cavities has been suggested to play a key role in Na^+^ ion mobility inside the crystal,^1,35^ while some studies suggest that the larger Cs^+^ ions need to be connected to open regions in decontamination applications.^37^ Defects have also been observed to have a key impact on transport properties, but the defect distribution is still not clear in PBA materials.^34,37,38^ Thus, it would be valuable to identify a transport mechanism that could provide a unified explanation of how ion diffusion depends on the local structure of the materials.

In this study, we have in detail investigated the key transport mechanisms in PBAs, including the impact of defects. The work starts with an investigation of the sodium-ion transport in Fe-based PBAs and continues with studies on potassium and other analogous monocations to give a broader perspective. We derive models over different dimensional scales including quantum chemistry to allow predictions of macroscopic performance based on atomistic insights. Furthermore, the model is employed to investigate differences between types of intercalating ions and PBA materials. Ultimately, the study seeks to deepen our understanding of intercalation transport and guide future development of materials.

## Theoretical Models

**Figure 2:**
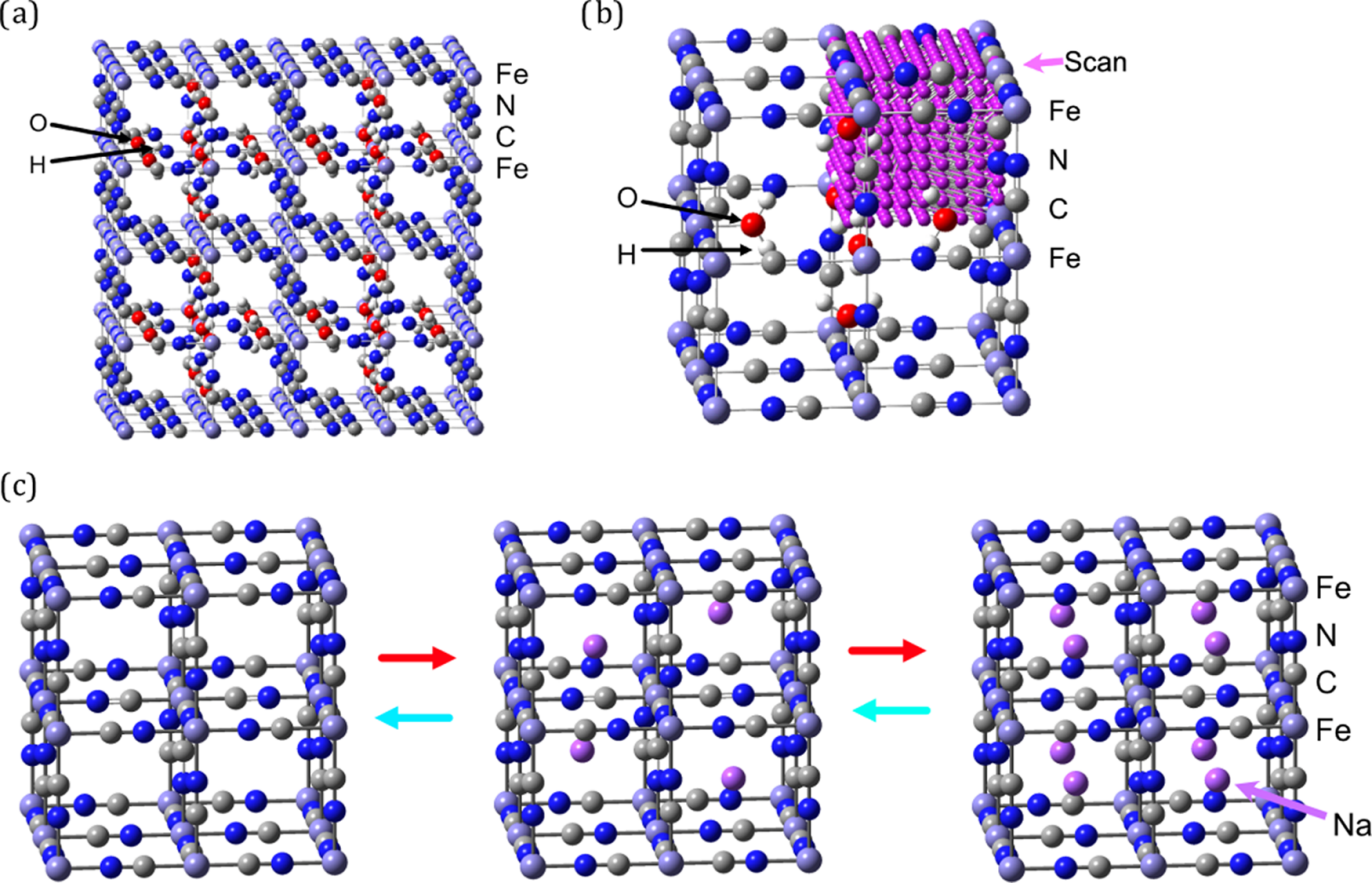
Crystal structure models used in the work. (a) 2 × 2 × 2 supercell of insoluble PB. (b) Energy scans of 9 × 9 × 9 points in one of the cavities in the PB unit cell. (c) Ideal PB unit cell. In distinct stages of charging, there are 0 (Berlin green), 4 (Prussian blue), or 8 (Prussian white) intercalating cations per unit cell.

The main atomistic simulations were based on the xTB program package for density-functional-based semiempirical calculations, as described by Grimme et al.^39^ and others.^40−42^ The model of Prussian blue was constructed using 2 × 2 × 2 supercells surrounded by periodic boundary conditions, as schematically represented in Figure 2a. The core structure in each cell followed the ideal structure of insoluble PB. The simulation included water that coordinated to Fe^3+^ near the central cavity but did not include additional zeolitic water in the structure (see, for instance, ref (37)). Periodic boundary conditions for the supercell further emulated a large crystal that was electroneutral with respect to the unit cells. Also, extra electrons were added to the crystal along with intercalating cations simulating charging in CDI experiments to preserve electroneutrality throughout all calculations.

To estimate the energy barriers of ion diffusion between cavities in the model structures, a script was written to include energies from xTB single-point calculations to probe a 9 × 9 × 9 mesh in one of the eight cavities in a unit cell of PBA (Figure 2b; see also Supporting Information, Figure S1). The single-point calculations generate the total energy of the system for a given set of atomic coordinates. Note that the valences of the ions are not a priori specified. This means that the differences in charges, such as between Fe^2+^ and Fe^3+^, are implicitly generated by the program based on the chemical conditions of the system studied.

Most simulations were based on the unit cell of a homogeneous, insoluble PB Fe_4_[Fe(CN)_6_]_3_·6H_2_O model structure,^4^ which contains a central vacancy with water coordinated to Fe^3+^ ions. To validate the results, we also included simulations on an ideal crystal structure without defects. An ideal PB structure exhibits three charged states (Figure 2c): the Berlin green (Fe^3+^[Fe^III^(CN)_6_]), the so-called soluble Prussian blue (AFe^3+^[Fe^II^(CN)_6_]), and Prussian white (A_2_Fe^2+^[Fe^II^(CN)_6_]).^4^ Intracavity charge is provided by intercalated cations A (such as Na^+^), while extra electrons are extracted from the environment (for instance, through the charging of the material connected to an electrode in an electrochemical device).

The diffusion constants were estimated using the package KineCluE,^43^ which is based on the self-consistent mean-field theory.^44^ The program utilizes Monte Carlo techniques to simulate the hopping between sites in a crystal as a basis to estimate the diffusion coefficient. At its core, the hopping rate Γ depends on the vibrational prefactor ν* (see eq 1)^45^ and the activation energy for the jump Δ*E*_b_.^44^ In this way, the approach can account for both how often an ion transfers between sites and how much time it spends in each site. In addition, in eq 1, *k*_B_ is the Boltzmann constant and *T* is the thermodynamic temperature.
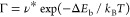
1

Macroscopic,^46−48^ and specifically finite-element modeling (FEM),^49−51^ can be relatively easily scaled up for simulating device-level performance. The macroscopic performance was simulated using the program suite COMSOL, based on Fick’s law for diffusion (eq 2).^52,53^ Here, *c* is the ion concentration inside the crystal and *D* is the diffusion constant. The macroscopic model was defined as one-dimensional with the same depth as a typical PB-crystal thickness used in experiments, where the material is considered to be homogeneous and symmetric across the length and width of the device.

The boundary conditions defining the end of the back contact was defined to have no flow (∂*c*/∂*x* = 0), while the border near the electrolyte was set to have a constant current (−*FD*∂*c*/∂*x* = *I*_0_, where *F* is the Faraday constant and *I*_0_ is the constant-current level). The voltage was estimated by assuming a simple linear relationship between the capacity and the voltage across the entire device. Finally, the circuit resistance of the device as a whole and the total storage capacity were determined in separate experiments and used as parameters in the model.
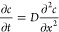
2

## Experimental Work

The simulation results were mainly benchmarked using data reported in the literature. Studies were selected based on providing data for both sides of the aspect property being compared. For instance, when comparing the intercalation locations of Na^+^ and potassium ions (K^+^), we refer to the earlier work that investigated this relationship experimentally. When data were included, they were extracted from graphs using WebPlotDigitizer^54^ software.

In addition, PB was synthesized during this work for experimental verification,^32,55^ as provided in the Supporting Information, including detailed characterization of the PB materials (Supporting Information, Figures S6–S8 and Table S1). The specific surface area and pore-size distribution of the prepared PB materials were evaluated in an Autosorb-iQ-MP-XR system using the multipoint Brunauer–Emmett–Teller (BET) and Barrett–Joyner–Halenda (BJH) methods, respectively (Supporting Information, Figures S6a,b).

To evaluate the conductivity and electrochemical properties, the synthesized PB materials were ground and mixed with a conductive additive (carbon black) and binder (PVDF) in the ratio 80:10:10. The mixture was prepared in *N*-methyl-2-pyrrolidone (NMP) to form a homogeneous slurry that was subsequently applied onto graphite sheets using a doctor-blade technique (1 × 1 cm^2^) and dried at 60 °C in a vacuum. The four-probe method using a CMT-SR1000N system was employed to record the electrical conductivity of the electrodes, and the results are shown in Table S1. The apparent diffusion coefficient of sodium ions in the PB materials was investigated by cyclic voltammetry (CV) with the ambition to enhance the accuracy of the simulated charging/discharging profile. The experiments were performed at different scan rates of 20, 10, 5, 3, 2, and 1 mV/s using a three-electrode setup in a supporting 1 M NaCl electrolyte solution via a Bio-Logic VMP3, France, electrochemical workstation (Supporting Information, Figure S7). Furthermore, the electrochemical kinetics of PB as the electrode material is represented by a Tafel plot in Figure S8 (Supporting Information).

The desalination performance was investigated using a typical CDI setup including PB and activated carbon (AC) electrodes and ion-exchange membranes.^55^ A constant-current mode with the current densities of 500, 200, 100, and 50 mA/g and a potential window of 1.2 to −1.2 V was employed using a battery analyzer (BTS4000, Neware) to evaluate the ion-intercalation behavior of the PB-based electrodes. In a batch-mode operation with 3000 ppm NaCl feed solution, a conductivity probe (DDSJ-308F, Leici) was placed near the outlet to monitor the in-line conductivity of the effluent. Other parameters, such as temperature (298 K) and flow rate (50 mL/min), were kept constant throughout the entire experiment. Sodium ions (cations) were intercalated into the PB lattice, while chloride ions (anions) were adsorbed to double layers on the activated carbon during charging.^16,55^ Conversely, during the discharging step, cations were deintercalated from the PB electrode material and the anions desorbed from the activated carbon electrode at the opposite side.

## Results and Discussion

### Core System

Using the results from the atomistic xTB-based calculations, we could simulate both molecular dynamics and explicit energy barriers. As a first step, the model accurately captures the diffusion and migration rates of sodium ions in water (Supporting Information, Figure S2). This suggests that the approach works well for investigating ion diffusion. Subsequently, a supercell of PB, corresponding to the structure in Figure 2, was constructed. The simulated lattice size agrees with experimental observations in the different charging states to within 1% (Supporting Information, Figure S3). This highlights that the program is relevant for investigating crystal structures, again without any preset or fitted calculational parameters. Having the validated core model of the PB system, it is now relevant to probe what diffusion should look like.

### Molecular Dynamics

The validated model system contains the PB crystal and sodium ions that can diffuse through it. To start the investigation with an open mind, we allowed the program to freely simulate the molecular dynamics to show how the system evolves (Supporting Information Video 1 shows a short movie of the dynamics of the system). The advantage of this approach is that it reveals how the system should behave naturally, with little impact from presumptions.

Video 1 reveals a surprising result. Intuitively, one might expect that ions inside a crystal would be pushed to cavities, vacancies, and other open positions. Here, the opposite seems to occur. None of the sodium ions enter the vacant region at the center of the unit cell. In fact, very few moves near the open cavities; instead, they pass diagonally between internal faces. This raises the question: why would the ions move in a way that is opposite to the normal intuition?

### Main Transport Pathway

Video 1 hints at an interesting mechanism for diffusive transport in PB. Now, a more systematic approach is required to understand the details. Thus, the first simulations probe the possible transitions inside the unit cell of insoluble PB and deduce the energy surface inside the unit cell.

**Figure 3:**
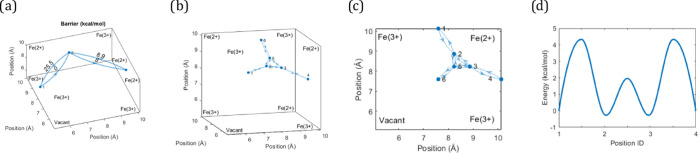
Transitions of Na^+^ in Fe_4_[Fe(CN)_6_]_3_·6H_2_O. The graphs show energy scans for a single cavity (1/8th of a unit cell). (a) These cumulative energy barriers are calculated as the sum of individual barriers along a path. The transitions correspond to pathways between the outer faces in the cavity or between the outer face and the inner face next to a vacancy. (b) Sites of minimum energy. (c) Top view of the sites in panel (b), showing that the local minima are closer to the wall than to the vacancy. (d) Energy landscape between the sites in panel (b). The reference levels are set to zero at the faces, while other minimum and maximum energy sites were calculated from the transition energies between the sites, averaged over all symmetric transitions. The line shows a cubic-spline interpolation for illustration purposes. These represent the transitions and energies that were used in KineCluE to calculate the diffusion constants.

In the complete energy scan, the 9 × 9 × 9 (Figure 2b) scan points make it possible to identify any possible transition through the cavity. The cavity cube is framed by six faces but only two symmetrically unique types of faces: those near the wall and those near the vacancy. Hence, the results show that two main types of transitions could allow an ion to move deeper into the crystal. Either the sodium ions can pass near the intact wall or they can pass near the defect center. Figure 3a shows the aggregated barrier for these two main transitions. The results suggest a striking difference in the aggregated energy barrier; the pathway close to the central defect requires 3 times higher energy of the transitions in comparison to that close to the cavity wall. This seems to verify the initial trends highlighted in Video 1.

Looking deeper into the transitions close to the wall, Figure 3b,c shows that Na^+^ displays local energy minima both on the internal faces of the cavity and at multiple sites in the cavity void. Interestingly, there are no local energy minima near the central defect and not even on the internal faces close to the defect. Moreover, the sites in the cavity void are shifted away from the central defect. Having confirmed the basic results, we can again ask: why would the energy strongly favor movement close to the wall rather than the open center?

Looking at the structure of PB, cyanide groups provide a negative charge density that attracts the positively charged sodium ions. Logically, it should therefore be energetically more favorable for the sodium ions to stay close to them rather than being alone in the void. This leads to a fundamental conclusion: to understand PB, we must think of diffusion in terms of attraction rather than repulsion. The ions are not repelled to the cavities but rather attracted to the walls.

Going deeper, the energies obtained suggest that there are two local energy minima along a diagonal path from frame to frame. Interestingly, all energy minima in Figure 3d have similar energies, suggesting that Na^+^ ions could populate any of these sites. This agrees well with the literature (where results from X-ray diffraction were reported).^4^ The transition path is not in a plane but rather curves around the Fe^3+^ atom at the corner of the unit cell. Because there are multiple barriers and local minima of similar energies along the path, a stepwise jumping mechanism could be of key importance for net ion mobility. From our newfound perspective of attraction, instead of repulsion being the driving force, it means that a good PBA material for fast diffusion should provide homogeneous attraction across the pathways.

The perspective of attraction also has implications for unit cells without a central defect. There will always be some unit cells of that type, created either by randomly clustered defect ordering or low-defect fabrication schemes. Having attractive groups surrounding the cation from all sides should stabilize it and raise the energy barrier for passing between cavities. Supporting simulations for a unit cell without a central vacancy verify this concept (Supporting Information, Table S2). However, the energy for passing an intact cavity is still much lower than that required to pass a vacancy. Thus, the vacant regions will act as effective barriers for diffusion. The next section will explore what this means for the diffusion pathways.

### Defining a Ladder Mechanism

The previous section highlighted the stark contrast between transitions near the intact wall and the central vacancy, which is a result of the attractive forces between the cation and the negatively charged cyanide groups that surround each internal face. We will henceforth use the word frame when describing the internal faces in PB to highlight the special properties derived from the cyanide groups. An intact frame is thus a frame in which all four surrounding cyanide groups are present and corresponds to the regions of fast diffusion. Near a vacancy, however, the frame is broken because the internal face is not covered by cyanide groups on all sides.

How would this finding relate to the diffusion pathway inside the crystal? Typical crystals contain a considerable density of defects; for instance, the typical insoluble PB Fe_4_[Fe(CN)_6_]_3_ is expected to miss at least 1/4 of [Fe(CN)_6_]. Similarly, for a +2 charge on the M ion and a +3 charge on the M′ ion, the M_3_[M′(CN)_6_]_2_ configuration should miss 1/3 of the sites. In principle, an added cation A could stabilize the structure and generate a defect-free AFe[Fe(CN)_6_] material but studies have shown that such configurations also display around 25% defects.^56^ This means that the defects, in general, must play a big role in the effective ion-diffusion dynamics in PBAs.

Recent studies have suggested that a nonrandom defect ordering can be present in PBAs.^34^ However, the mean-field simulations highlight that the diffusion coefficient for transitions that pass a defect site is lower by around 10 orders of magnitude, as compared to the ideal transitions modeled as well as experimental findings. This means that ion transport near defects cannot constitute the most probable transition, and there should be some variation in how the defects are distributed in these experiments involving adsorption/desorption in PBAs.

**Figure 4:**
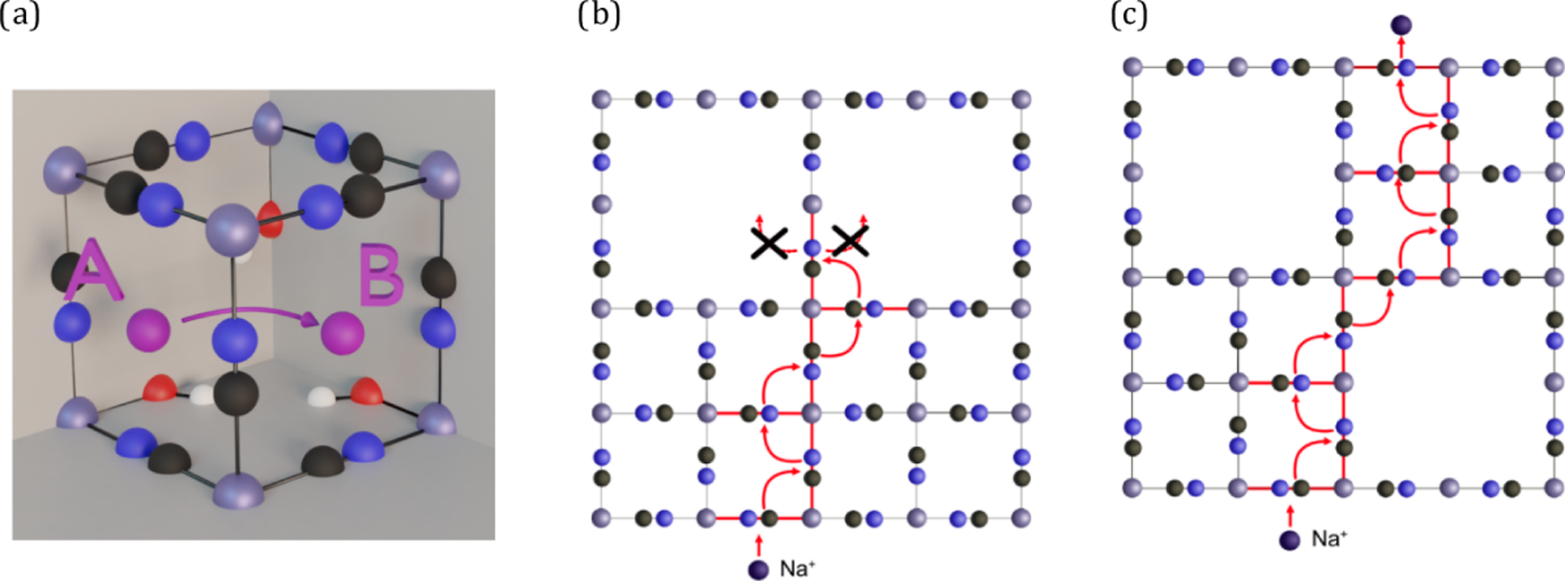
Illustrated transitions near defects. (a) Graphics show a single cavity out of the eight in the unit cell (see the unit cell in Figure 1). In a cavity, Na^+^ ions (pink) can diagonally move from face to face (A to B) in three dimensions. (b) Projected 2D view of the transitions in a supercell with defects. If two defects are side-by-side, there is no way for the ion to move diagonally past the defect void. (c) As long as there is a diagonal pathway, the ions can move deeper into the material.

To summarize, we can denote frame-mediated diffusive transport in the defect-rich environment as a ladder mechanism (Figure 4a). Previous studies have noted that the negatively charged CN^–^ skeleton is essential for facilitating the Na^+^ transport^1,35^ because the ions need these accumulations of negative charge to generate sites inside the crystal, between which the cations can jump. However, the simulation results here go one step further, and we notice that the four CN^–^ groups on a face create a framework that must be intact to facilitate the Na^+^ movement. In analogy, a ladder is broken if either the steps or the rails are missing. Similarly, if an [Fe(CN)_6_] complex is missing, it ruins the structure of all of the eight adjacent frames (Figure 4b) and thus the facilitating ladders of ion diffusion. Still, as long as the frames are intact, the Na^+^ ions can move diagonally from frame to frame (Figure 4c). Moreover, as long as there is a diagonal pathway, the ions can move deeper into the material. However, the defects will still create bottlenecks of high energy barriers, which limit the number of effective pathways. Thus, the overall defect density and organization must affect the macroscopic diffusion constant of the material.

### Effect of the Defect Concentration

Knowing the qualitative effect that the defects should have on the diffusion pathway, can we predict how the defect concentration may affect the macroscopic diffusion rate? Since PBAs are characterized by high densities of defects, the ordering of the defects will be important for determining the availability of channels for ion transport (Figure 4b) or if a majority of pathways will form dead ends (Figure 4a). Previous studies have typically assumed random defect distributions in PBAs,^37,38^ and we will adopt that assumption here too, and we, therefore, propose a formulation for estimating the impact of defects on the overall diffusion.

Every face borders two M′ ions in the [M′(CN)_6_] complex and each of the M′ coordination centers could be missing (holes in Figure 4b,c). Hence, if the probability that a position is filled (nondefect) is *p*_f_^2^, then the probability that a face will be intact is *p*_f_^2^. Analogous to a porous material, this means the permissible volume is only the fraction ϵ_p_ = *p*_f_^2^ (ϵ_p_ is equivalent porosity). Aside from reducing the transport volume, this also means that the ions must pass a mazelike (tortuous) path to go deeper into the material. According to the Bruggeman model,^52^ the effective diffusion in such a material would be represented by eq 3. In eq 3, *D* is the diffusion constant, *D*_e_ is the effective diffusion constant, and τ is the tortuosity.
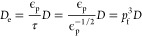
3By direct computation, comparisons with experimental data in the literature reveal that eq 3 is adequate for predicting the variations in effective diffusion with respect to different defect densities^1,35^ (Table 1). This suggests that a random model of defect distribution will be representative of the typical PBAs reported in the literature.^1,35^ To test the derived expressions for the effect of random distributions, we also constructed a 13 × 13 × 13 supercell using MATLAB and simulated the effect of randomly distributed defects on the distribution of intact faces and the resulting variation in an effective diffusion constant (Supporting Information, Figure S9). The simulations validate the expression in eq 3 for a random defect distribution.

**Table 1:** Relative Diffusion Constants^a^

property	pred.	ref (1)	ref (35)
*D*_rel_ = *D*_L_/*D*_H_	0.36	0.10	0.45

a These are the predicted (this work) and experimentally determined (refs (1) and (35)) relative diffusion constants for Fe PB. Note that each of the references used crystals with two different qualities, high and low, corresponding to the presence of few or many defects, respectively. The value in the table is the relative diffusion constant *D*_L_/*D*_H_ from each reference offering a platform for comparison. The simulation was carried out assuming 6 and 33% defects in high- and low-quality crystals, respectively.^35^

The results also show that while higher defect densities are always detrimental to ion diffusion, changes in defect densities show a much greater impact if the defect densities are low, as indicated by the *p*_f_^3^ term in eq 3. Thus, for instance, purifying a crystal from a 5% defect density to 0% makes a substantial difference, while going from 25 to 20% defects leads to a much smaller difference in cation diffusion. Also, because the ions preferably travel via intact transport channels, clustering of the defects would allow a higher density of efficient ion pathways and therefore lead to an overall improvement in transport properties of the material.

From the fundamental concept of the ladder mechanism, we have thus been able to zoom out one step and predict the variations in an effective diffusion constant. Now, the next question is: is it possible to zoom out one step further and describe the device-level performance?

### Macroscopic Perspective

So far, in all of the above simulations, we have obtained energies or relative energies, and as a consequence, the results are mainly qualitative. This makes it interesting to investigate the consequences the difference in activation energy will have on the macroscopic transport of the intercalated ions. Using a mean-field theory approach, we simulated how Na^+^ would move in the energy landscape, as described in Figure 3c. The method accounts for jumping energies, residence time, and attempt frequencies at the intercalation points to estimate the total diffusion constant for the material. The obtained diffusion coefficient for pure transitions (1.9 × 10^–12^ m^2^/s) is higher than commonly reported experimental values, as apparent diffusion coefficients are typically determined by cyclic voltammetry (1.08 × 10^–12^ or 1.46 × 10^–14^ cm^2^/s in this work, which is similar to refs (1, 35)). More importantly, the simulated diffusion coefficients for pathways passing a vacancy are around 10 orders of magnitude lower than for transitions along unperturbed pathways, meaning that defects cannot constitute a realistic pathway for ion transitions. Thus, the quantitative simulations verify the transport principles found in the previous sections.

To further investigate the relevance of the obtained diffusion constants, we constructed a 15 μm thick crystal of iron PB (Fe[Fe(CN)_6_]) and investigated the device performance (the electrode was 15 μm thick including binders and conductive additives). Using cyclic voltammetry to investigate two samples of PB, the diffusion coefficient was estimated to be 1.08 × 10^–12^ cm^2^/s (Supporting Information, Figure S9, single crystal) and 1.46 × 10^–14^ cm^2^/s (Supporting Information, Figure S10, PB grown on a cloth consisting of activated carbon). These results show clear similarities with previously reported values for PB.^1,35^ More importantly, this suggests a diffusion coefficient highly similar to that obtained from modeling—i.e., that the energy landscape in the material can be generalized and is relevant for a variety of materials prepared by different techniques.

**Figure 5:**
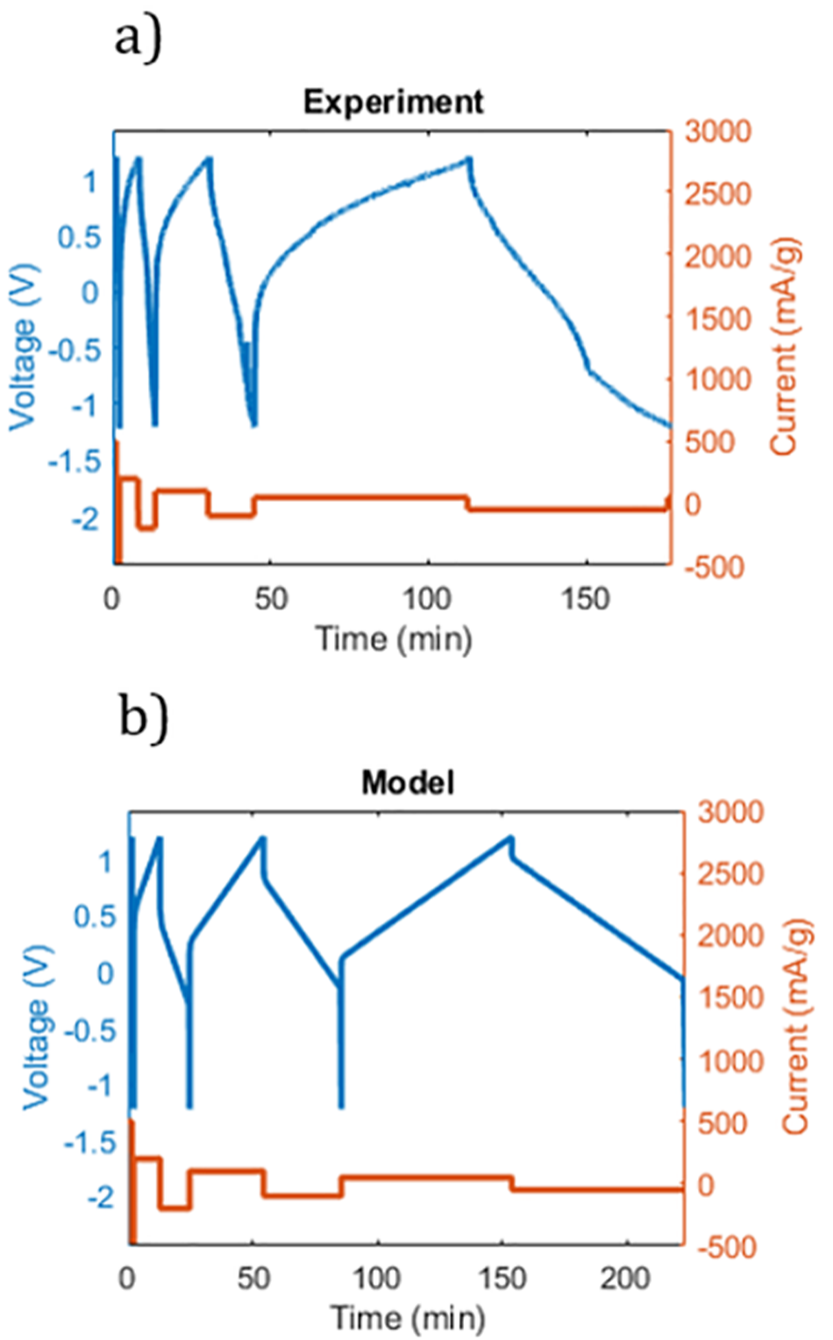
Voltage profile during charging and discharging of a device containing Fe PB as the electrode material. The counter electrode used consisted of a large mass (“overdose capacitance”) of activated carbon. The constant-current cycles were based on 500, 200, 100, and 50 mA/g for both charging and discharging. (a) Experimental voltage and current. (b) Model-predicted voltage and current.

The experiment was performed with different constant-current charging rates with a cutoff voltage at 1.2 V (Figure 5). The results show that the total storage capacity of the device is substantially higher at lower charging rates, which suggests that the longer time for diffusion allows the ions to diffuse to a larger part of the material volume. The Supporting Information, Table S3, shows how much Na^+^ is intercalated depending on the charging rate for the electrodes studied.

The simulations were based on Fick’s law of diffusion combined with the calculated diffusion coefficient, and the performance shows a similar trend with respect to the relationship between diffusion and total capacitance. This result is striking, considering that detailed simulations at an atomistic level have been used to predict macroscopic device-level performance. It is also notable that the vertical jumps in the voltage (Figure 5b) correspond to the total electrical resistance and demonstrably contribute substantially to the variation in the capacitance depending on the charging rate.

The slow transport kinetics inside the intercalation materials has means so that their capacity can increase for longer charging times, as the ions have time to transport farther into the bulk. The simulations above show that it is possible to use atomistic-level simulations to predict the impact of also ion-transport kinetics on a macroscopic scale via a multiscale theoretical approach.

The findings in this section have shown that the ladder mechanism can be used to understand both the fundamental mechanism and at the same time describe the macroscopic performance. The following sections will take a step back and investigate how well these results generalize to the wider family of PBA materials and intercalating ions.

### Other Ions

The previous section shows that detailed atomistic models can, when combined with FEM, simulate macroscopic device performance. Interestingly, the atomistic model offers these results without introducing any fitted parameters at the device level. Having developed this multiscale model for ion-transport properties, a deeper question arises regarding the general validity for various intercalating cations and material compositions in the PBA family.

**Figure 6:**
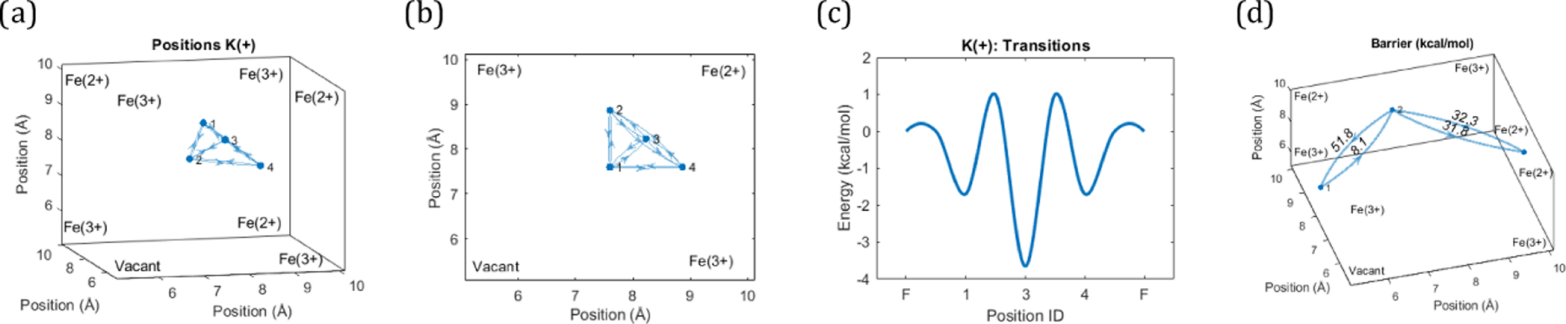
Transition energies for ions other than Na^+^, specifically, K^+^ and Fe^3+^. (a) Position of the energy minima of K^+^ in Fe PB. (b) Top view of the sites in panel (a). (c) Transition energies for the energy minimum sites in panel (a) together with the energies at the faces (denoted as F). The energy at the walls is defined as the zero level, while the minimum and maximum energies are determined by the transition energies, averaged over all symmetric transitions. (d) Transition energies for a few noteworthy transitions of Fe^3+^. Also, the transition for an Fe^3+^ escaping from the crystal matrix backbone into the cavity (not shown) has an energy above 600 kcal/mol.

Starting with K^+^, simulations show that this ion displays less pronounced energy minimum sites in the PB cavities in comparison with Na^+^ (Figure 6). This difference between the ions agrees with previous reports based on crystal structures.^4^ The simulations show that the energy minimum sites for the larger K^+^ ions are shifted away from the cavity walls more toward the space in the cavity center.

Indeed, a scan of the energy landscape for the energy minimum sites and the face sites reveals that there is a single energy well right between the cavity walls. The depth of this well indicates that the simulated activation energy for K^+^ is similar but slightly larger than that for Na^+^. This implies that their diffusion coefficients should be similar, which agrees well with our experimentally estimated diffusion coefficients based on electrochemical impedance spectroscopy (EIS) (Supporting Information, Figure S10).

The Supporting Information shows a similar energy scan for Li^+^ (Figure S11). All of the investigated ions display the same behavior of preferred transport pathways via the faces in contrast to passing the vacancies. This suggests that the ladder mechanism holds for all of the investigated ions.

For even larger intercalated ions, the energetically favorable positions shift further and further toward the empty void close to a defect. As the energy of the central well decreases, the energy barriers at the faces become so large that they slow or block ion transport. At the extreme, the transition energies for the Fe^3+^ matrix ions are too massive for transport between faces and much larger still for leaving the matrix of the material backbone. This suggests that the material as a whole is unlikely to break down spontaneously or form more defects without any significant external influence.

Studies in the literature often discuss the transport of different ions in terms of the ionic size and compare the size of the ion with the size of the facework space.^37^ Looking at sizes alone, the central position in the cavity should be the intercalation site, while the face is the most energetically difficult place to traverse. However, the results above highlight that the interactions between the ion and the material facework are the most important because they determine the energy landscape through which the ions move. For instance, sodium ions had lower energy in the “wall” than in the cavity because the negatively charged CN^–^ groups attract the positively charged ions. Also, Figure 6 for K^+^ could either be interpreted in terms of higher energies in the wall or lower energies in the central transition states.

A crucial general experience is that all of the simulated ions show the same fundamental behavior: negative attracts positive, and therefore it is always more favorable for a cation to stay closer to the wall than spend time close to the vacancy. As shown in Figures 3 and 6, activation energies and intercalation positions will vary, but the ladder mechanism holds for all of the investigated ions.

### Prussian Blue Analogues

Because PBA is a wide family of materials, another central question is how generally applicable is the ladder mechanism to the various analogues. Mainly, variations in the energy landscape could affect the activation energies and hence the overall diffusion constants. Therefore, we simulated the transitions for PBA materials containing divalent M = {Fe, Co, Cu, Mn} to identify a qualitative difference in host behavior regarding intercalated ion diffusion. Note also that the previous work has suggested that the M ion shows a greater impact than the M′ ion in the [M′(CN)_6_] complex for the centrosymmetric structural stability of the materials.^34^

**Figure 7:**
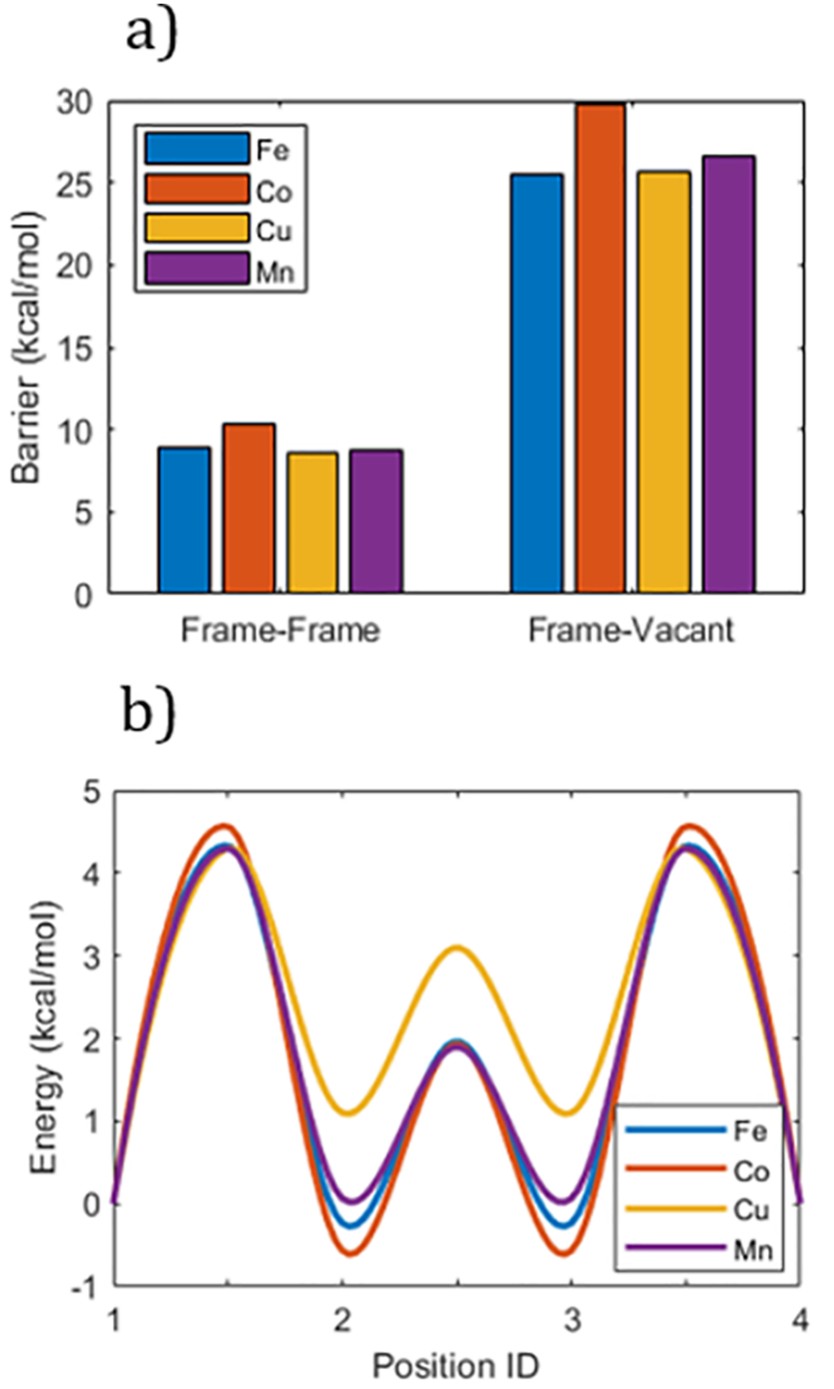
Simulated energy landscapes for a variety of PBAs. The materials contain M = {Fe, Co, Cu, Mn} combined with the [Fe(CN)_6_] complex. (a) Position IDs correspond to the face and body positions in Figure 3b. The energy at the faces is defined as the zero level, while the minimum and maximum energies are determined by the transition energies, averaged over all symmetric transitions. Note that the energy is “per transition” and 1 kcal/mol = 0.043 eV. (b) Aggregated barrier for (diagonal) frame–frame transitions and transitions via the vacancy.

To start, Figure 7a shows that the difference in the energy barrier is huge independent of the PBA material. Also, the frame-vacant transition is described by one huge barrier, while the smaller frame–frame transition consists of smaller transitions. This means that the actual difference in the diffusion rate should be even larger than that indicated by the figure. The main conclusion is that the ladder mechanism holds for all of the investigated PBA materials. The reason for this is that any differences from the material are negligible as compared to the inherent energy barriers for distancing the diffusing cations from the negative sites. Thus, the proposed ladder mechanism is robust with respect to changes in the material’s conditions.

Looking deeper into the frame–frame transitions, the results suggest that the difference in the activation energy for exiting the face minima in various materials is insignificant (Figure 7b). However, there is a clear difference in the energy levels at the intermediary sites between the faces. On the one hand, this shifts the most likely position to find a Na^+^ ion between the face and the cavity void. On the other hand, this also changes the probability that the ion will get stuck at the metastable intermediary sites while traveling between the face energy minima.

Notably, PBA materials containing Cu show the most shallow energy wells at the intermediary sites, suggesting faster diffusion rates of the resulting PBA material, and not surprisingly Cu has been reported to enhance structural^34^ and cycling^4^ stability. Since the curves shown in Figure 7 illustrate the relative energies with respect to sites 1 and 4, the reasons for the seemingly more shallow part of the curve arising when Cu^2+^ is included can be a bit ambiguous. However, if assuming that the Cu^2+^ interacts less strongly with the CN^–^ ligands than the other divalent metal ions studied, the resulting negative partial charges on the CN^–^ ligands may be slightly higher and therefore for mainly electrostatic reasons generate energetically lower minima for the sites 1 and 4. As a result, the section of the curve involving sites 2 and 3 will appear more shallow.

In this study, we have learned that the ladder mechanism of ionic transport is generally applicable to PBAs and various intercalating ions. Also, the previous section showed that a key difference in the transport performance between ions originates from the relative energies between the face and the central transition sites. This section showed that the material composition can affect the relative energies between the face and the central transition sites. Thus, future materials research and theoretical studies could benefit from investigating how to smoothen the diagonal transitional landscape for specific intercalated ions.

### Sensitivity Analysis

The core simulations of this work are based on atomistic calculations at a semiempirical level and without device-level fitted parameters for determining the diffusion. Thus, it is relevant to look at how well the model system corresponds to reality and specifically how variations in the model output will influence the final results.

Generally, the semiempirical calculations have been found to be quite accurate for simulating transport properties. For instance, the predicted values for free diffusion and electromigration of Na^+^ ions correspond well to the experimental values (Supporting Information, Figure S2).

For the nonsubstituted, Fe-based PB crystal, the simulated optimal lattice parameter is also close to the value reported in the literature (Supporting Information, Figure S3). The simulations that are based on the crystal structure with a central vacancy thus underestimate the noncharged material unit-cell size by about 1%. Overall, the unit-cell size dependence on the charging state is close to experimental observations, with differences around 1% between the noncharged and fully charged states (Supporting Information, Figure S3). Notably, PB has wide lattice spacing and the difference in the unit-cell size from charging has a minor impact on the energy for the intercalated ion transitions (Supporting Information, Figure S4). This is different from some lithium-ion systems, where expansion can be well over 100% and thus substantially impact diffusion.^57^

A point to note is that if there already is an ion present inside a cavity, the energy barrier for the entry will be high (Supporting Information, Figure S5). Such electrostatic blockages could locally slow the overall diffusion. However, the ions will still be able to move freely into deeper empty cavities, thus such blockages are expected to only affect the results marginally.

Another similar feature that could slow diffusion is the presence of interstitial water molecules. Water can be abundant in PBA. For instance, Takahashi et al. synthesized various forms of K*_y_*Cu[Fe(CN)_6_]_1–*x*_·*z*H_2_O.^37^ They found that the defect concentration *x* determined the other proportions in terms of *y* = 4 – 2*x* and *z* = 10*x*. This corresponds to every unit cell with a central vacancy (Figure 1) containing 10 water molecules (6 coordinated and 4 interstitial ones). In our work, we have only considered the coordinated water molecules. However, experiments have suggested that interstitial water both reduces the storage capacity and the diffusion rate of the cations.^1,57−59^ This is reasonable if water molecules block the pathways or partially hydrate the cations. Here, nothing suggests that water molecules should facilitate pathways via the vacancies, and therefore, the conclusion regarding the ladder mechanism should not be affected by the interstitial water content.

The calculated diffusion coefficients depend exponentially on the activation energy. While this means that a small error in the activation energy will make a huge difference in the estimated diffusion constants, the noise variation within the simulations corresponds to less than 1 order of magnitude in the variation of the estimated diffusion constant. Thus, the macroscopic calculations are be expected to show some variations with the conditions used, but these variations are expected to be sufficiently small to ensure that the results are experimentally relevant.

More crucially, the simulation noise is small enough to guarantee that the validity of qualitative comparisons between different transitions, different cations, and different substituted PB atoms are relevant. For instance, the predicted diffusion coefficient for the vacancy transition is 10 orders of magnitude lower, suggesting that even errors on the order of 1 magnitude in the absolute diffusion coefficient would not change the conclusions based on the simulation results. We also verified the substantial difference in the activation energy between the pure and vacant transitions (Figure 3a) using a reaction-path method.^60^

The quantitative analysis suggested that the experimentally determined diffusion coefficient in PB is significantly lower than that simulated based on a diagonal transition in a structurally intact unit cell. Because the vacant transport is much too slow to constitute an important pathway, this suggests that there could be another bottleneck to diffusion that ions do pass. Investigations of unit cells with and without central vacancies suggest that the diffusion is substantially slower in an intact unit cell without a central vacancy because the surrounding CN^–^ groups stabilize the cation from all sides. For K^+^, such a higher energy barrier (Supporting Information, Table S2) yields a diffusion coefficient of 3.3 × 10^–15^ cm^2^/s, which is within 1 order of magnitude of the experimentally determined value (Supporting Information, Figure S10). While diagonal transitions in unit cells are fast, this would imply that ions cannot go deeper into a material without passing either a vacancy or an intact region, hence yielding a slower overall diffusion rate.

### Outlook

The multiscale approach developed in this work has several similarities and differences when compared to the earlier work on other conducting systems. These have been essential for probing the specific nature of the ladder mechanism in PBAs.

Various approaches exist for estimating energy barriers. In the work by Takahashi et al.^37^ on Cs adsorption in PB, the authors calculate the diffusion barriers by simulating the energies in assumed positions for intercalation sites and wall sites. Similar approaches are also used for other crystal types. For instance, Zhuang et al.^57^ calculated the potential energy surface for the diffusion of Li in metals by scanning the positions between the start and end sites. The current work takes a different approach because we recognize that the defects can create locally complex intercalation landscapes. Complex intercalation landscapes can be important for intercalation positions, as well as the transition paths, and they can have a substantial impact on the performance of the material.^61^ Thus, the scans in this work cover the entire volume that the ions can move in. This is key to both identifying the intercalation sites and the main transition pathways in the complicated geometry of PB.

The energy landscape found highlights the importance of investigating the local interactions for understanding if transitions are favorable. An interesting comparison can be found in the work by Zhao et al.^62^ where the authors discovered that rotational motions in some solid electrolytes can help to deliver lithium ions between stable sites. Thus, both the favorable sites and the fastest routes between them can be crucial for understanding the diffusion in complex materials.

The possible effects of defects are interesting from several perspectives and can vary between materials. Wang et al.^63^ showed that substitutional defects in bcc Fe can become traps that hamper diffusion of O. On the contrary, Shadike et al.^64^ noted that antisite defects of 1/6 Cr/V′Na in Na_0.5_CrS_2_ can have a positive effect on diffusion. In the current work, we observe that the open regions completely block diffusion and give rise to the ladder mechanism. On the other hand, the diffusion rate at the corner of a vacant unit cell is faster than in a unit cell without a vacancy. This is because cavities that enclose ions from all sides provide more stability to the intercalation site.

Another interesting aspect of the current multiscale approach is the link between the quantum-chemistry simulations and FEM. Some earlier studies focus mainly on comparing energy barriers and executing parameter scans for macroscopic transport.^57^ Using the self-consistent, mean-field method for simulating ion hopping, we are instead able to generate a unique prediction for the macroscopic diffusion constant based on the energy levels obtained from the quantum-level simulation. Also, every new level of simulation brings new challenges. Thus, our current work combines the quantum-level output with a macroscopic prediction of tortuosity to estimate the effects of the defect density. Future work could build on this method on different scales. For instance, quantum-level simulations can be used to estimate intercalation energies,^61^ and expanded FEM calculations can be used to simulate larger device-level performance.

## Conclusions

This study is based on a multiscale modeling approach applied to the ion-transport mechanisms of intercalated cations in PBA materials. The atomistic simulations reveal a ladder mechanism of cation transport. The intercalated cations move diagonally between internal faces in unit-cell cavities that are surrounded by four negatively charged CN^–^ groups (frames). The ions need these CN groups as ladder steps to climb inside the crystal, but a ladder is broken unless it has both steps and rails. In an analogy, the intercalating ions get support from all of the surrounding frames of CN^–^ groups. If a vacancy ruins part of this frame, the entire passage in essence becomes impermissible to the moving intercalation ions. When transporting diagonally from frame to frame, the intermediary local energy minima are identified as crucial sites for the cation transport properties. Based on a model of random defect distribution, we have also derived an accurate and simple description of the impact of defects on the overall diffusion constant.

Based on the energy barriers obtained with simulations at an atomistic level, we further used self-consistent mean-field theory to estimate the effective diffusion coefficient. Subsequently, by coupling the effective diffusion coefficient with FEM calculations, a multiscale model that combines all dimensional scales is completed to allow simulations of device-level performance.

The differences in transport properties of PB-intercalated Na^+^ and K^+^ differ mainly in the internal coordination site they preferably occupy. Specifically, the larger K^+^ ions show energy minima that are shifted away from the internal walls of the PB cavities. Larger ions, such as Fe^2+/3+^, show activation energies of mobility too large to permit diffusion across internal material cavities. When comparing different PBA materials, the simulation indicates that a selection of PBAs (divalent M = {Fe, Co, Cu, Mn}) exhibits similar activation energies in the frame to frame transitions. However, the energy levels at the intermediary points differ significantly, and as a consequence, some materials will show a tendency to trap ions in the cavities and thus slow ion transport.

## Supporting Information Available

Transition data before postprocessing (Figure S1); performance of xTB for free diffusion and migration (Figure S2); lattice size from charging (Figure S3); simulated energies with a different lattice size (Figure S4); transition energies with a blocked interstitial site (Figure S5); BET characterization of the PB electrode (Figure S6); conductivity measurements for the PB electrode (Table S1); cyclic voltammetry measurement (Figure S7); Tafel plot for the PB electrode (Figure S8); relationship between the intact frames and vacancy ratio (Figure S9); material characterization of PB nanoparticles (Figure S10); comparison of energy barriers between transition types (Table S2); and relationship between the current density and electrode capacity (Table S3) (PDF)
Film of molecular dynamics (Figure S11): main transitions for lithium in PB (MP4)
