# Supplementary material for: Ladder Mechanisms of Ion Transport in Prussian Blue Analogues
Source: ACS Appl Mater Interfaces. 2021 Dec 22;14(1):1102–13. doi: 10.1021/acsami.1c20910 (PMC8762639; doi:10.1021/acsami.1c20910)
Supplement: Supplementary file 1 — am1c20910_si_001.pdf [file am1c20910_si_001.pdf]

Supporting Information

Ladder Mechanisms of Ion Transport in Prussian Blue Analogs

Johan Nordstrand *[1], Esteban Toledo-Carrillo [1], Sareh Vafakhah[2], Lu Guo[2], Hui Ying Yang[2], Lars Kloo[3], Joydeep Dutta [1]

[1] Functional Materials, Applied Physics Department, School of Engineering Sciences, KTH Royal Institute of Technology, AlbaNova universitetscentrum 106 91 Stockholm, Sweden

[2] Pillar of Engineering Product Development, Singapore University of Technology and Design, Singapore 487372

[3] Applied Physical Chemistry, Department of Chemistry, KTH Royal Institute of Technology, SE-100 44 Stockholm, Sweden

*Corresponding author. Email: johanno3@kth.se

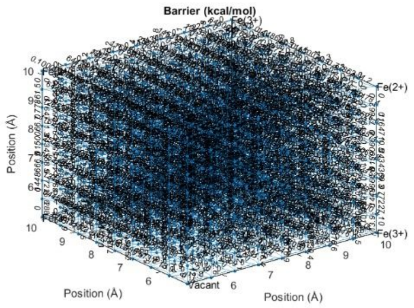

Figure S1: All transitions before postprocessing. The point here is the show that the scan searches all possible transitions in a 9x9x9 grid to find the transition paths within the crystal.

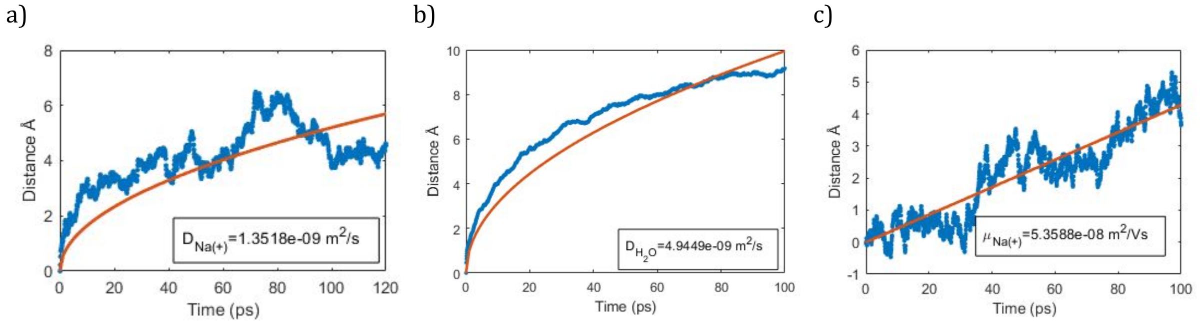

Figure S2. The performance of xTB for free diffusion and migration. The point here is to verify that the semi-empirical calculations from xTB manage to replicate the diffusion and electro-migration properties of sodium and water. (a) Diffusion of Na+ in water. (b) Diffusion of water in water. (c) Diffusion migration of Na^+ in water under an applied electric field.

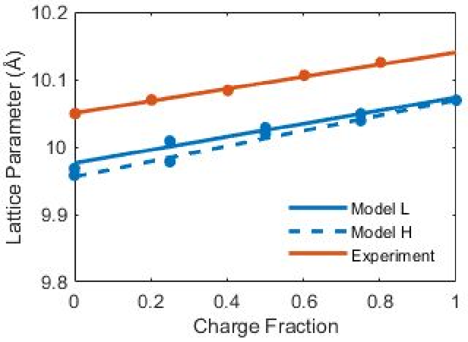

Figure S3: Lattice size from charging (Cu-PB with K^+ ions). This graph shows that the semi-empirical calculations replicate the trend of increased lattice spacing in PB, without fitting parameters. The experiment data is from Ref. [4]. Model L (low quality/many defects) corresponds to Cu-PB with the charging of 0-4 K+ ions per unit cell. Model H (high quality/few defects) corresponds to Cu-PB with the charging of additional 0-4 K^+ per unit cell. In both cases, the program calculated the lattice size by scanning the total energy for a range of sizes and selecting the size corresponding to the lowest energy.

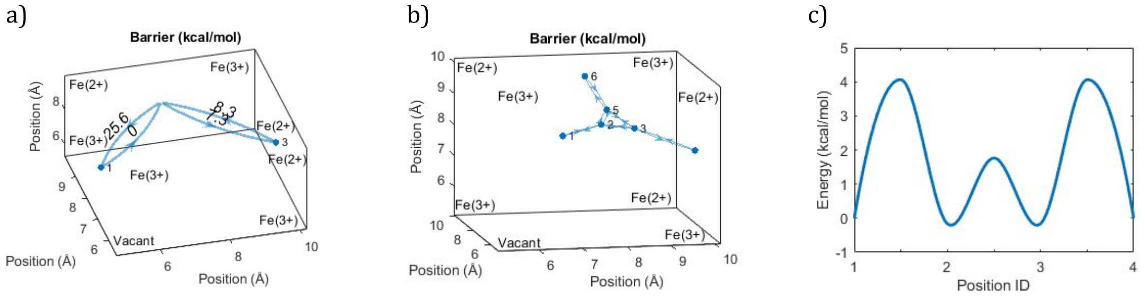

Figure S4: Simulated energies with a crystal size different from that in the main manuscript by 1 %; that is, the approximate difference between the simulated and experimentally optimal size in Figure S3. (a) The cumulative energy barriers between intercalation sites at the internal faces of the cavity. (b) The energy minima that were found in the scan. (c) The energy landscape in (b). Note that the line shows a cubic interpolation between points of maximum and minimum energies, as calculated with xTB.

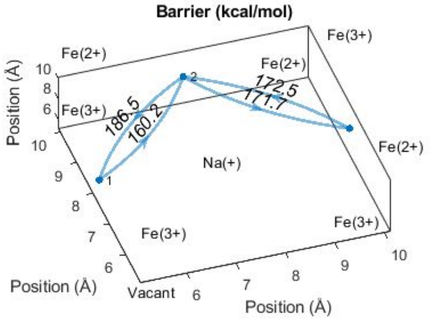

Figure S5: The energy for transitions when there is another Na^+ present in the cavity. The high energies indicate that a Na^+ ion can block incoming Na^+ ions.

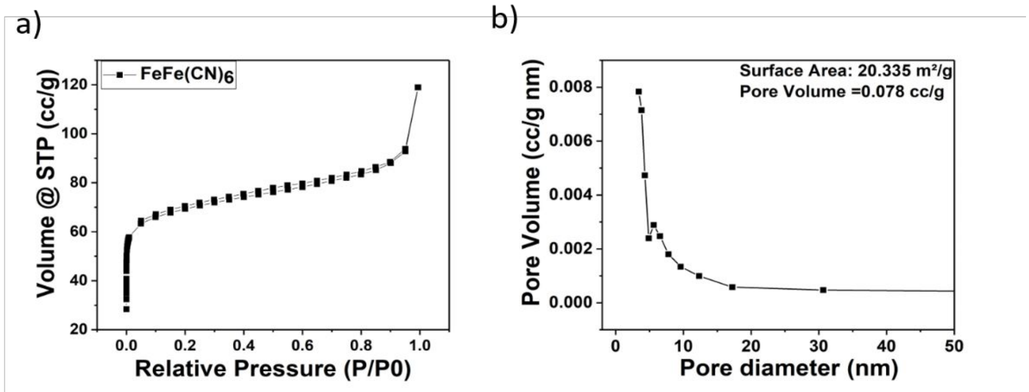

Figure S6. BET characterization of the PB electrode used in Figure 5, showing the surface area and pore volume.

Table S1. Conductivity measurements for the Prussian Blue electrode pasted on graphite sheet including PB, Carbon Black, and Binder with the ratio of 80:10:10.

**Points**	**Resistance (ohm)**	**Conductivity (μS/cm)**
**1**	0.01668	140
**2**	0.01455	160
**3**	0.01509	154
**Avg**	0.01544	151

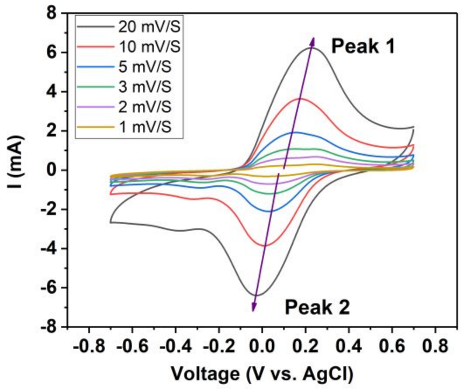

Figure S7. The raw data for the cyclic-voltammetry measurement for the PB electrode at different scan rates.

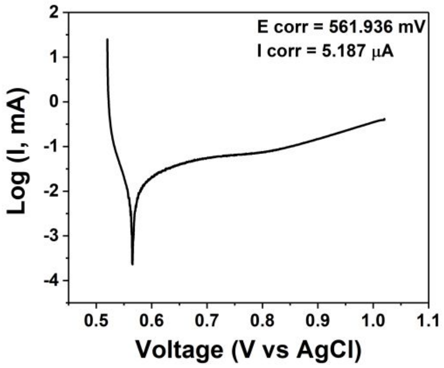

Figure S8. The Tafel plot and the corresponding E corr and I corr of PB electrode that is used in Figure 5.

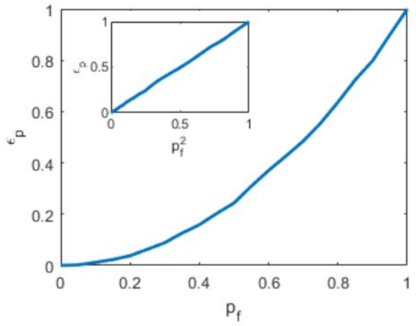

Figure S9. Main image: The simulated fraction of intact frames ϵ_p as a function of the probability p_f that a site for $Fe(CN)_6$ is vacant. The simulation used a 13x13x13 supercell of iron Prussian blue, where the vacant sites are randomly selected from all $Fe(CN)_6$. Inset: With the same data as in the main image, this graph demonstrates that the trend between p_f and ϵ_p is quadratic, as predicted in Equation 3.

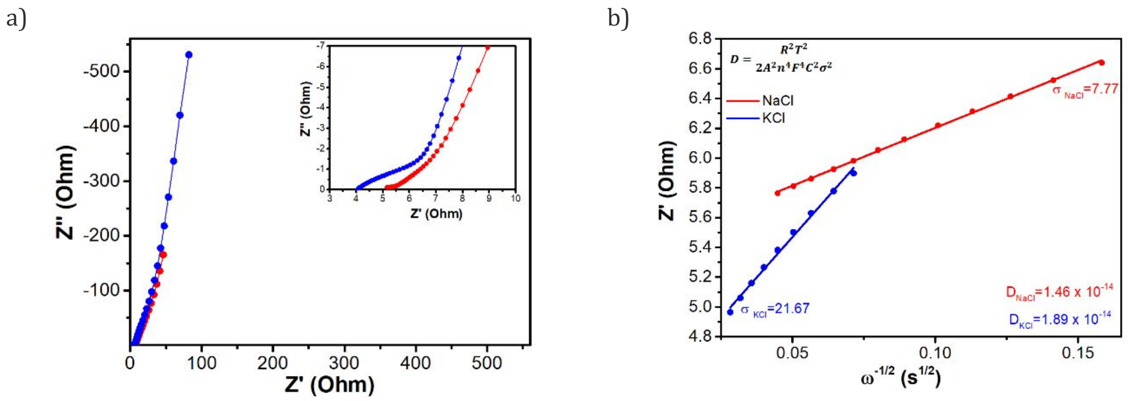

Figure S10. Material characterization of PB nanoparticles grown of activated carbon cloth (not the same sample as Figure S8). (a) EIS spectra. (b) Calculated apparent diffusion coefficients of Na^+ and K^+ (NaCl and KCl solutions) in the material.

Table S2: Comparison of energy barriers from transitions in unit cells with and without a central defect. Here, cumulative barrier denotes the sum of barriers along with a path between two stable positions. The Frame-Frame and Frame-Vacant are the same transitions as in Figure 3a.

Transition Type of K^+ in Cu-PB	Cumulative Barrier (kcal/mol)
**Frame-Frame**	7.4
**Frame-Frame (no Vac.)**	15.8
**Frame-Vacant**	34.5

Table S3. The relationship between current density in sodium-ion storage capacity for the sample of PB used in **Error! Reference source not found.Error! Reference source not found.**. Note that the capacity denotes mg NaCl per g of PB.

Current Density (mA/g)	Capacity (mg/g)
**500**	9.44
**200**	18.1
**100**	29.5
**50**	51.16

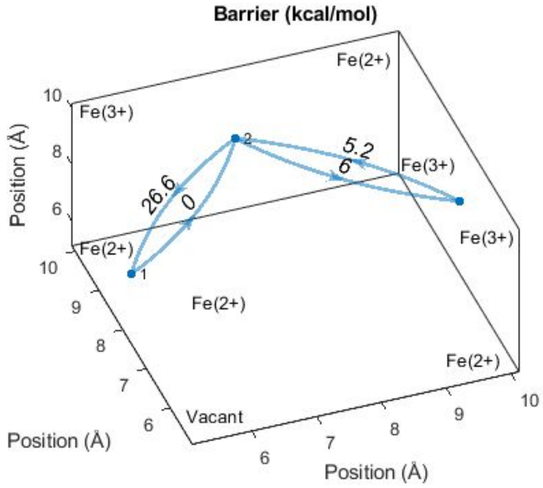

Figure S11: Transition energy of Li^+ in Fe-PB for transitions between the faces of the cavity.
